# Therapeutic potential of amino acid-based peritoneal dialysis solutions: a systematic review

**DOI:** 10.1186/s12882-026-05222-3

**Published:** 2026-07-30

**Authors:** Bengt Lindholm, Antoine Barbari, Jennifer Allen, Inès Dufour, Donald Fraser, Annette Heider, Rumeyza Kazancioglu, Monika Lichodziejewska-Niemierko, Anabela Malho-Guedes, Loris Neri, Alena Parikova, Juan Carlos Quevedo-Reina, Adonay Santana-Quintana, Udaya Udayaraj

**Affiliations:** 1 Renal Medicine, Karolinska Institutet, M99 Karolinska University Hospital Huddinge, Stockholm, 14186 Sweden; 2 Renal and Transplant Unit, Rafik Hariri University Hospital, Beirut, Lebanon; 3 Renal and Transplant Unit, Nottingham University Hospitals NHS Trust, Nottingham, UK; 4 Division of Nephrology, Cliniques Universitaires Saint-Luc, Brussels, Belgium; 5 Wales Kidney Research Unit, School of Medicine, College of Biological and Life Sciences, Cardiff University, Cardiff, UK; 6 Department of Nephrology, Klinikum Neumarkt and KfH Neumarkt, Neumarkt, Germany; 7 Division of Nephrology, Bezmialem Vakif University Faculty of Medicine, Istanbul, Türkiye; 8 Department of Nephrology Transplantology and Internal Medicine, Medical University Hospital of Gdańsk, Gdańsk, Poland; 9 Serviço de Nefrologia, Unidade Local de Saúde do Algarve, Faro, Portugal; 10 Renal and Dialysis Unit, Michele e Pietro Ferrero Hospital, Verduno, CN Italy; 11 Department of Nephrology, Transplant Center, Institute for Clinical and Experimental Medicine, Prague, Czech Republic; 12 Servicio de Nefrología, Hospital Universitario de Gran Canaria Doctor Negrín, Las Palmas, Spain; 13 Oxford Kidney Unit, Churchill Hospital, Oxford, UK; 14 Nuffield Department of Medicine, University of Oxford, Oxford, UK; 15 Department of Palliative Medicine, Medical University of Gdańsk, Gdańsk, Poland

**Keywords:** Peritoneal dialysis, Amino acid-based solutions, Glucose-based peritoneal dialysis solutions, Glucose sparing, Clinical outcomes, Adverse events

## Abstract

Peritoneal dialysis (PD) offers many advantages over hemodialysis but chronic exposure to bio-incompatible glucose-based PD solutions and continuous absorption of glucose from the dialysate may lead to peritoneal membrane (PM) dysfunction and systemic metabolic complications such as hyperglycemia, insulin resistance, dyslipidemia, and obesity. Replacing one daily exchange of 1.36% glucose-based solution with one 1.1% amino acid-based PD solution may provide nutritional benefits of amino acid supplementation, improve biocompatibility, and reduce the peritoneal glucose load while maintaining similar ultrafiltration and solute removal capacity. Randomized clinical trials and mechanistic studies show beneficial effects of amino acid-based PD solutions on peritoneal homeostasis, preserved viability of the PM, and favorable nutritional and metabolic effects. This systematic review highlights the clinical rationale for implementing use of amino acid–based PD solutions as an integral part of glucose-sparing strategies in PD to reduce the cumulative intraperitoneal glucose load and mitigate its well-documented adverse effects. Amino acid–based PD solutions emerge as a clinically viable, glucose-free alternative and a nutritionally beneficial option that can improve the nutritional status of PD patients by providing essential amino acids when used as part of a comprehensive glucose-sparing strategy. Targeted use of these solutions, particularly in malnourished or high-risk patients, represents a personalized approach to optimizing peritoneal dialysis outcomes.

**Clinical trial number:** Not applicable.

**Supplementary Information:**

The online version contains supplementary material available at 10.1186/s12882-026-05222-3.

## Highlights

**What was known:**

Replacing one daily exchange of 1.36% glucose-based solution with one 1.1% amino acid-based PD solution addresses the abnormal amino acid pattern in patients with kidney failure and reduces the peritoneal glucose load while maintaining similar ultrafiltration and solute removal capacity. Supplementation of essential amino acids may provide nutritional benefits and reducing peritoneal glucose exposure may improve biocompatibility. However, a comprehensive systematic review of clinical studies of amino acid-based solutions has been lacking.

**This study adds:**

This systematic review highlights the therapeutic potential of amino acid-based PD solutions in addressing protein-energy wasting by providing an alternative nitrogen source while at the same time reducing glucose exposure. The evidence synthesized suggests that one daily exchange of a 1.1% amino acid solution is generally well-tolerated and effective in improving nutritional surrogate markers such as serum albumin and nitrogen balance. However, significant heterogeneity remains in the literature, and long-term data on technique survival and mortality are still lacking.

**Potential impact:**

Targeted use of these solutions, particularly in malnourished and high-risk patients, represents a personalized approach to optimizing nutritional status and may reduce potential adverse local and systemic effects of peritoneal glucose exposure. Further studies are warranted to establish long-term efficacy of the 1.1% amino acid solution.

## Background

Peritoneal dialysis (PD) remains an essential and effective home-based renal replacement therapy for patients with end-stage kidney disease (ESKD) [1, 2]. Although global utilization rates have fluctuated over time and, in some regions, experienced a relative decline [3], PD continues to provide several clinically relevant advantages over hemodialysis (HD), including superior preservation of residual kidney function (RKF), greater patient autonomy and quality of life, and lower infrastructural requirements [1, 2]. Despite advances in technique and survival rates, long-term PD use is limited by complications such as peritoneal membrane (PM) dysfunction, metabolic complications, and technique fatigue [4].

A key contributor to the shortened time on PD therapy is structural changes of the PM over time, characterized by fibrotic thickening and vascular alterations [5–9]. These changes lead to functional changes that compromise ultrafiltration (UF) and solute clearance, especially as RKF declines—a factor independently associated with higher mortality [10].

Emerging evidence implicates conventional PD solutions in these deleterious effects [11]. Standard formulations, typically acidic and hyperosmolar due to high dextrose content, contribute to membrane toxicity and patient discomfort [12–17]. Thermal sterilization of these solutions generates glucose degradation products (GDPs), which promote the formation of advanced glycation end products (AGEs), further inducing chronic peritoneal damage, including fibrosis, neovascularization, and increased solute transport [12, 18–24]. These alterations impair local immune responses, increasing peritonitis risk [25–27], and GDP absorption may also directly contribute to RKF decline [8, 28, 29].

Another problem with glucose-based PD solutions is that they may contribute to metabolic complications due to the continuous uptake of glucose from the dialysate [2]. The advent of non-glucose osmotic agents in PD regimens to limit glucose exposure and its associated metabolic burden is therefore a major advancement [12–17, 30–32].

Amino acid-based PD solutions, offer less glucose exposure, with osmotic properties comparable to 1.36% glucose solutions [30, 31, 33, 34]. These solutions have been approved for once-daily use and are being used as part of glucose-sparing strategies in PD [30, 31].

This systematic review aimed to evaluate the efficacy and safety of amino acid–based peritoneal dialysis solutions (unless otherwise specified represented by Nutrineal PD4, Vantive, former Baxter Healthcare Corp., Deerfield, IL, USA). The goal is to inform best practices for integrating amino acid-based PD solutions into glucose-sparing dialysis regimens.

For this purpose, we scrutinized evidence from clinical trials, observational studies, and mechanistic research to assess the impact of amino acid–based PD solutions on PM structural and functional properties including UF capacity, and metabolic tolerance. Additionally, adverse events were examined and compared to outcomes associated with conventional glucose-based solutions.

## Methods

### Search strategy and eligibility criteria

A comprehensive literature search was conducted across PubMed, Medline, Embase, and Google Scholar to identify randomized controlled trials (RCTs) and real-world evidence (RWE) studies evaluating the efficacy and safety of amino acid–based PD solutions up to November 30, 2025.

The search strategy incorporated Medical Subject Headings (MeSH) and keyword combinations, including: “Peritoneal Dialysis” OR “Peritoneal Dialysis, Continuous Ambulatory” AND “amino acid-based” OR “amino acid-based peritoneal dialysis solutions” OR “Nutrineal.” Additional free-text searches were performed in titles and abstracts using terms such as Continuous Ambulatory Peritoneal Dialysis (CAPD), Continuous Cycling Peritoneal Dialysis (CCPD), Automated Peritoneal Dialysis (APD), kidney failure, and peritoneal dialysis in various combinations.

To ensure comprehensiveness, reference lists of included studies were manually screened, and relevant articles were also identified based on expert recommendations.

The current study included RCTs and retrospective and prospective RWE studies conducted on patients with chronic kidney disease (CKD) who underwent treatment with PD and for which the efficacy and/or safety outcomes were reported.

This inclusive approach allowed for a broad evaluation of the clinical impact of amino acid-based dialysates in PD.

The search included only studies published in English, French, Portuguese, Italian, or Spanish.

The Preferred Reporting Items for Systematic Reviews and Meta-Analyses (PRISMA; http://www.prisma-statement.org/) guideline was established to enhance the transparency and completeness of systematic review reporting [35, 37]. Nonetheless, it exhibits certain limitations, particularly in offering detailed recommendations for data synthesis and result presentation [35–38]. Furthermore, substantial heterogeneity across studies often hinders quantitative aggregation of findings [36]. Narrative reviews, while commonly used, also present significant methodological challenges, including insufficient transparency, inadequate methodological rigor, and poor disclosure of review limitations [39, 40].

Due to the significant heterogeneity in study designs, patient populations, and reported outcomes across the identified literature, a formal grading of the level of evidence for each individual study was not performed. Instead, we utilized the Synthesis Without Meta-analysis (SWiM) reporting guideline to provide a transparent qualitative synthesis of the available data in contexts where quantitative aggregation was deemed inappropriate.

The SWiM approach has been developed to guide systematic reviews in contexts where meta-analytical techniques are either inappropriate or unfeasible [39]. Notably, nearly one-third of intervention-focused systematic reviews in health research do not incorporate a meta-analysis [39]. This systematic review was conducted without meta-analysis and adhered to the methodological framework provided by the PRISMA guidelines [41].

### Study selection and data extraction

The authors independently generated the queries for the literature search and selected the articles fulfilling the criteria established for each subject and solved any disagreement through discussion and consensus. To determine the eligibility, searched papers, including title, abstract, and full text were evaluated.

The data that were extracted included information about the study; efficacy outcomes, and safety outcomes (incidence and type of adverse events).

Data extraction was conducted independently by two investigators using a standardized electronic template. For each study, we recorded the author, year of publication, study design, geographical location, patient characteristics, and primary clinical or mechanistic outcomes. In cases where data were presented solely in graphical form, numerical values were extracted using WebPlotDigitizer (Version 4.7. 2026. Available from: https://automeris.io/WebPlotDigitizer) to ensure accuracy. Discrepancies between reviewers were resolved through consensus. The extracted data were managed in Microsoft Excel and categorized by clinical application to support the qualitative synthesis.

## Results

### Search results and study characteristics

**Fig. 1:**
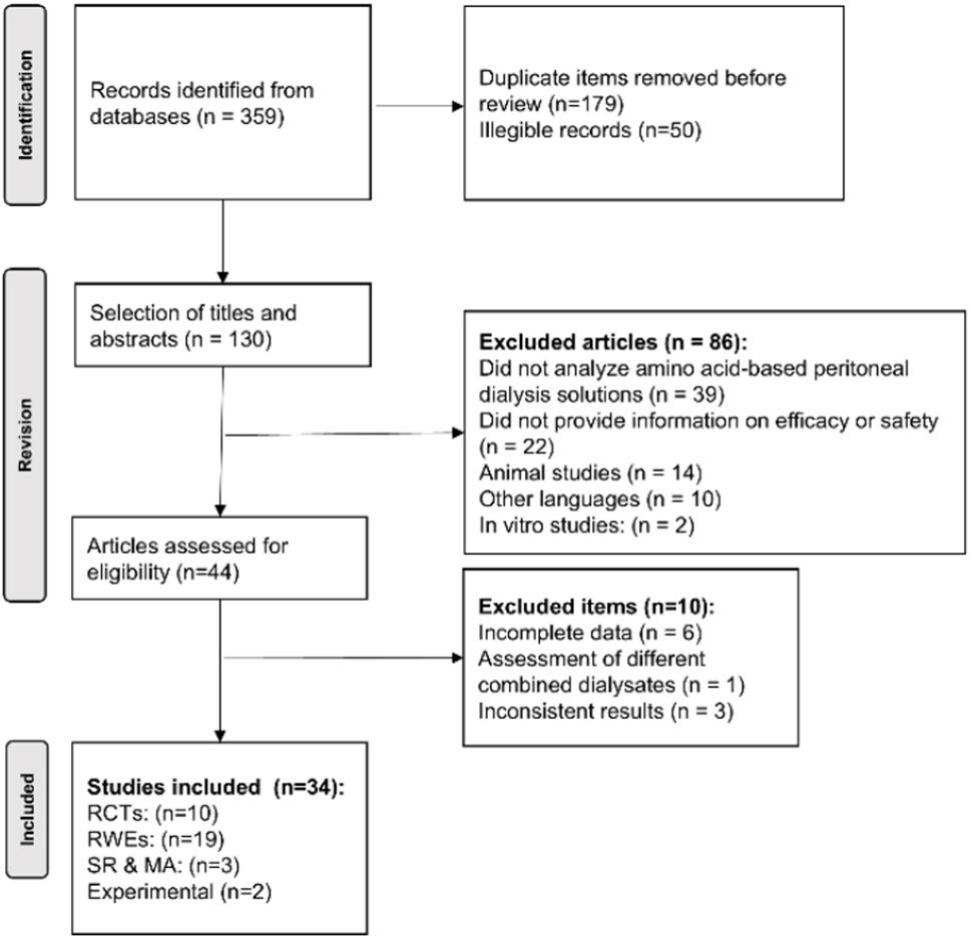
Flowchart of reporting elements referred to for systematic reviews and meta-analyses (PRISMA). RCTs, randomized controlled trials; RWEs, real-world evidence studies; SRs, systematic reviews; MAs, meta-analyses

The flow chart of the selection process is shown in Fig. 1. A total of 359 articles were identified through database searching. After removing duplicates, 130 articles were evaluated by their titles and abstracts. Out of these, among 44 studies that met the criteria for a full-text review, 10 were excluded due to incomplete data, assessment of different combined dialysates, and/or different outcomes. Finally, a total of 34 papers, 10 RCTs, 19 RWE studies, two experimental studies, and 3 systematic reviews, were eligible for the qualitative analysis (Fig. 1; Table 1).

**Table 1:** Summary of included studies on amino acid–based dialysates in peritoneal dialysis. Articles are listed according to their year of publication

Author / Year	Design	Country	Objective	Main Outcomes
Kopple et al. 1995 [42]	Experimental	Multicenter	Assess effects of AA-based PD fluid on protein nutrition	Improved nitrogen balance, net protein anabolism, plasma amino acids, serum total protein, and transferrin
Misra et al. 1996 [43]	RCT	UK	Assess nutritional effects of Nutrineal in CAPD patients	Improved nitrogen balance and serum albumin
Douma et al. 1996 [44]	Real-world	Netherlands	Examine effect of amino acid dialysate on peritoneal blood flow	Increased peritoneal blood flow; suggested NO-mediated mechanism
Misra et al. 1997 [45]	RCT	UK	Examine effect of AA dialysate on lipid metabolism	No adverse lipid profile changes; nutritionally beneficial
Steele et al. 1998 [46]	Real-world	Singapore	Case study in hypoalbuminemic vegetarian patient	Improvement in serum albumin with IPAA use
Jones et al. 1998 [47]	RCT	USA	Evaluate replacement of AA/protein losses using Nutrineal	Improved protein balance; no adverse metabolic effects
Martis et al. 1998 [48]	Real-world	USA	Overview of new PD solution composition	Theoretical and early clinical support for AA-based solutions
Jones et al. 1998 [49]	RCT	Multicenter	Assess effects of AA-based PD solution	Significant increase in MAMC from baseline; no change in peritoneal membrane transport characteristics
Delarue et al. 1999 [50]	Real-world	France	Evaluate protein balance (Leucine metabolism)	Improved protein synthesis in CAPD patients with no effect on protein breakdown.
Plum et al. 1999 [51]	Prospective, randomized, cross-over.	Germany	Investigate the acute metabolic responses and peritoneal transport behavior	Increased of plasma amino acids; decreased in serum glucose vs. glucose-based fluids; positive nitrogen balance; no change in ultrafiltration, solute transport, or acid–base status.
Chung et al. 2000 [52]	Review	Sweden	Discuss biocompatibility of newer PD solutions	Emphasized potential of AA-based fluids in reducing bioincompatibility
Weryński et al. 2001 [53]	Real-world	Sweden	Kinetic comparison of dipeptides vs. AA-based PD fluids	Showed comparable solute kinetics and favorable absorption
Taylor et al. 2002 [33]	Real-world	UK	Assess long-term Nutrineal use in hypoalbuminemic patients	Increased albumin and protein catabolic rate
Li et al. 2003 [54]	RCT	Hong Kong	3-year study on Nutrineal in CAPD patients	Improved serum albumin, cholesterol, and dietary protein intake
Chan et al. 2003 [55]	Real-world	Hong Kong	Examine mesothelial ultrastructure under different dialysates	AA solutions less damaging than glucose-based fluids
Chang et al. 2003 [56]	Real-world	Taiwan	Assess homocysteine metabolism with AA-based dialysate	No significant adverse changes; safe metabolic profile
Voges et al. 2004 [57]	Real-world	Belgium	Evaluate stability of drug additives in PD solutions	Confirmed compatibility in new container types
le Poole et al. 2004 [58]	RCT	Netherlands	Compare low-glucose vs. standard PD regimens	Improved metabolic parameters and patient tolerance
Mortier et al. 2004 [59]	Real-world	Belgium	Assess peritoneal membrane effects of new PD solutions	AA-based solutions reduced markers of fibrosis
Reimann et al. 2004 [60]	Real-world	Germany	Study NO production by mesothelial cells at exposure to AA-PD	Increased NO release with AA-based solution
Tjiong et al. 2005 [61]	RCT	Netherlands	Evaluate combined AA + glucose dialysate effects on protein anabolism	Increased protein synthesis; positive nitrogen balance
Martikainen et al. 2005 [62]	Real-world	Finland	Investigate inflammation and peritoneal preservation with glucose-free solutions	Reduced inflammatory markers; membrane preservation
Duranay et al. 2007 [63]	Real-world	Turkey	Compare peritonitis rates across dialysate types	Lower rates with biocompatible/AA-based solutions
Tjiong et al. 2007 [34]	RCT	Netherlands	Assess AA + glucose dialysate during feeding	Enhanced protein synthesis during oral intake
Tjiong et al. 2007 [64]	RCT	Netherlands	Compare effects of oral vs. intraperitoneal AAs on protein synthesis	Distinct metabolic pathways; IP AA more anabolic
Chang et al. 2007 [65]	Real-world	Taiwan	In vitro effects of glucose-free solutions on mesothelial cells	Less cytotoxicity with AA-based fluids
Dervisoglu et al. 2010 [66]	Real-world	Turkey	Evaluate the influence of 1.1% amino acid dialysis solutions on nutritional parameters in incident CAPD patients.	No significant changes in serum albumin levels. No differences between the AA-based and glucose-based solutions.
Yung et al. 2015 [67]	RCT	Hong Kong	Evaluate fibrosis and inflammation markers in low-glucose PD	AA-based regimens showed reduced fibrotic markers
Kussmann et al. 2015 [68]	Real-world	Austria	Assess antibiotic activity in various PD fluids	Compatibility maintained in AA solutions
Rusai et al. 2013 [69]	Real-world	Austria	Investigate protective mechanisms in mesothelial cells	GSK-3β inhibition with AA solutions supported cell survival
Tobudic et al. 2017 [70]	Real-world	Austria	Evaluate antibiotic compatibility with PD fluids	Fosfomycin stable in AA solutions
Kussmann et al. 2017 [71]	Experimental	Austria	Evaluate fosfomycin compatibility with PD fluids	Fosfomycin was stable in AA solutions
Iyasere et al. 2022 [72]	Systematic Review & Meta-analysis	Multicenter	Assess anthropometric changes with AA dialysate	Small but consistent improvements in nutritional parameters
Ling et al. 2023 [73]	Systematic Review	Multicenter	Evaluate antibiotic compatibility in PD solutions	Confirmed broad compatibility including AA-based fluids

RCT: Randomized control trial; RWE: Real-world evidence; AA: Amino acids; PD: Peritoneal dialysis; CAPD: Continuous ambulatory peritoneal dialysis; IP: Intraperitoneal; MAMC: Midarm muscle circumference; NO: Nitric oxide

A total of 34 studies were included in the systematic synthesis. Of these, 28 studies provided extractable quantitative data concerning nutritional parameters, metabolic outcomes, or antibiotic stability. The remaining six studies, comprising three secondary reviews, one clinical overview, one case study, and one qualitative ultrastructural analysis were analyzed qualitatively to provide mechanistic depth and contextual support for the discussed clinical recommendations.

### Amino acid–based peritoneal dialysis solutions

Following the introduction of amino acid-based peritoneal dialysis (AA-PD) solutions in 1968 as a nutritional supplement, serving as an alternative to conventional glucose-based PD solutions [74], several investigators examined the nutritional benefits of substituting amino acids for glucose in peritoneal dialysis solutions [30, 31, 42]. Current amino acid–based PD fluids containing 1.1% amino acids provide an osmotic gradient equivalent to 1.36% glucose solutions while eliminating intraperitoneal glucose exposure and its metabolic sequelae [75]. This solution does not contain dextrose or icodextrin, using the amino-acid mixture as the osmotic agent. Its electrolyte profile is essentially the same as standard PD fluids [76, 77]. These solutions are available for use in both CAPD and APD. In APD, they may serve as the last fill in the morning (to be drained by midday to prevent fluid overload) or be administered during nocturnal dialysis mixed with glucose [34].

Nutrineal PD4 is a 1.1% solution containing 15 amino acids (total: 11.1 g/L) and standard electrolytes (Na⁺, Cl⁻, Ca²⁺, Mg²⁺, and lactate as a bicarbonate precursor), see Table 2. Its osmolarity (~ 365 mOsm/L) and pH (~ 6.6) are comparable to conventional PD solutions [76–80].

**Table 2:** Composition of Nutrineal PD4 amino acid-based peritoneal dialysis solution [54, 76–80]

Essential amino acids, mg/L		Semi-essential amino acids, mg/L	
Histidine	714	Tyrosine	300
Isoleucine	850		
Leucine	1020	Non-essential amino acid, mg/L	
Lysine	764^a^	Alanine	951
Methionine	850	Arginine	1071
Phenylalanine	570	Glycine	510
Threonine	646	Proline	595
Tryptophan	270	Serine	510
Valine	1393		
Total amino acids		Electrolytes, mmol/L	
%	1.1	Calcium	1.25
w/v/gL	11.1	Magnesium	0.25
		Sodium	132
Amino acids, mmol/L	87.2	Chloride	105
		Lactate	40
pH	6.2-6.7	Osmolarity, mOsm/L	365

w: weight; v: volume

^a^Lysine hydrochloride 955 mg/l

Calcium chloride dihydrate 184 mg/l; Magnesium chloride hexahydrate 51 mg/l; Sodium chloride 5380 mg/l; Sodium lactate 4480 mg/l

The absorption of amino acids from AA-PD varies according to the PM’s transport characteristics, which are typically classified based on the dialysate-to-plasma creatinine ratio (D/P₍Cr₎) obtained during a 4-hour peritoneal equilibration test (PET) using a high concentration glucose solution. ideally 3.86% [81–85]. These classifications include high (fast) peritoneal solute transfer rate (PSTR) (D/P₍Cr₎ > 0.80), high-average PSTR (D/P₍Cr₎ between 0.65 and 0.80), low-average PSTR (D/P₍Cr₎ between 0.50 and 0.64), and low (slow) PSTR (D/P₍Cr₎ < 0.50) [81–85].

Patients with high PSTR, characterized by D/P₍Cr₎ > 0.80, exhibit rapid amino acid absorption due to increased membrane permeability. However, this rapid absorption can lead to a swift dissipation of the osmotic gradient, potentially reducing UF efficiency over longer dwell times [81–85]. For this reason, CAPD-based schemes are particularly suitable for low transporters, while APD-based schemes are more appropriate for high transporters [25, 29]. Conversely, patients with low (slow) PSTR, with D/P₍Cr₎ < 0.50, absorb amino acids more slowly, but prolonged dwell times can enhance both amino acid absorption and UF. Patients with high-average PSTR and low-average PSTR display intermediate absorption rates, maintaining a balance between solute clearance and UF, making them suitable candidates for standard PD regimens [81–85].

### The rationale behind glucose sparing peritoneal dialysis solutions

Glucose-sparing strategies in PD have been developed to mitigate the adverse effects associated with conventional glucose-based PD solutions. Traditional PD fluids utilize high concentrations of glucose as the primary osmotic agent, which can lead to systemic metabolic complications such as hyperglycemia, insulin resistance, dyslipidemia, and obesity, particularly in diabetic patients [6, 8].

In addition, prolonged exposure to glucose-rich solutions poses significant risks, including formation of GDPs, structural and functional PM damage, and systemic metabolic complications such as insulin resistance and cardiovascular disease [8, 12–14].

Non-glucose-based osmotic agents such as icodextrin and amino acids, provide an alternative glucose-sparing approach, to counteract the unfavorable effect of glucose and GDP exposure on PM and cardiometabolic system. Icodextrin, a glucose polymer, provides sustained UF over long dwell times and has been shown to improve fluid management and reduce cardiovascular risk factors in PD patients [86].

Clinical studies have demonstrated that the combination of non-glucose-based solutions such as amino acid-based agents and icodextrin into PD treatment regimens can reduce systemic glucose exposure and improve the systemic metabolic profile in PD population [12–14, 17].

### Amino acid-based peritoneal dialysis solutions and the peritoneal membrane

PM injury during PD arises from both intrinsic and extrinsic factors, triggering a sustained inflammatory response that disrupts the mesothelial cell (MC) layer and impairs membrane integrity. Chronic exposure to bioincompatible PD solutions can lead to mesothelial denudation, fibrosis, and loss of peritoneal function. Detached mesothelial cells attempt repair by producing growth factors and extracellular matrix proteins, but persistent inflammation can overwhelm these mechanisms, resulting in structural and functional deterioration of the PM [87]. This pathological cascade promotes neoangiogenesis, fibrosis, and ultimately leads to UF insufficiency, a major cause of PD technique dropout [88, 89].

The MC monolayer, which serves as the initial interface with dialysis fluids, is particularly vulnerable to bioincompatible factors such as GDPs and AGEs. These compounds contribute to the activation of epithelial -to-mesenchymal transition (EMT), a process by which MCs lose their epithelial polarity, adopt a mesenchymal phenotype, and begin producing excessive extracellular matrix and pro-angiogenic mediators [85].

Given its central involvement in fibrotic remodeling and vascular changes of the PM, EMT represents a critical pathogenic mechanism and a promising therapeutic target for preserving peritoneal function during long-term PD [90].

Conventional glucose-based PD solutions exert local toxicity on the PM, largely due to their acidic pH and the generation of GDPs during heat sterilization and prolonged storage. GDPs contribute significantly to peritoneal damage by inducing oxidative stress, stimulating inflammatory responses, and promoting angiogenesis and apoptosis in various peritoneal cell types [91–93].

A key pathological mechanism involves the formation of AGEs, which are derived from GDPs such as 3-deoxyglucosone (3-DG). These AGEs interact with their cellular receptor (RAGE), amplifying fibrotic, angiogenic, and proinflammatory signaling pathways that accelerate PM deterioration [94, 95]. Notably, it has been shown that exposure of MCs to GDPs alone, even in the absence of glucose, is sufficient to trigger AGE accumulation and a strong inflammatory response [93]. Furthermore, Igaki et al. demonstrated that 3-DG—not glucose itself—plays a central role in late-stage protein glycation, highlighting the distinct cytotoxicity of specific GDPs independent of glucose concentration [96].

Overall, these findings underscore the critical role of GDPs and AGEs in driving structural and functional damage of the peritoneal membrane, positioning them as central targets for improving the biocompatibility of PD fluids [88, 89].

AA-PD solutions, such as Nutrineal PD4, are formulated with a more biocompatible profile than conventional acidic glucose-containing solutions and were developed to prevent or treat malnutrition while minimizing patient exposure to glucose and its degradation products, which contribute to peritoneal membrane injury [14–17, 52, 97, 98]. By limiting glucose load, these solutions generate fewer glucose degradation products, offering potential advantages for membrane preservation, although their acidic pH may still pose concerns. These solutions exhibit enhanced biocompatibility compared with conventional glucose-based fluids, inducing less cellular stress, preserving mesothelial cell viability, and maintaining cytokine production, indicative of reduced proinflammatory responses and improved membrane integrity [51, 99, 100]. Bicarbonate buffering further supports acid-base homeostasis, reinforcing their favorable profile for long-term peritoneal membrane protection [51, 99, 100].

Clinical investigations support this premise. In a study by Douma et al. [44], AA-PD significantly enhanced peritoneal blood flow and solute transport compared with glucose-based dialysate. Among stable CAPD patients, a 4-hour dwell with AA-PD increased carbon dioxide mass transfer area coefficient (CO₂-MTAC), a surrogate for blood flow, from a median of 60 to 93 mL/min (*p* < 0.01). Additionally, MTACs for small solutes including creatinine, urea, and urate were significantly elevated [44]. Although transcapillary UF was transiently higher with AA-PD, the net UF over 4 h was comparable due to a compensatory increase in lymphatic absorption. Importantly, the use of AA-PD did not substantially increase the clearance of larger molecules; albumin and immunoglobulin G (IgG) losses were only marginally greater during the AA-PD dwell compared to glucose-based dialysate [44].

Cellular studies provide mechanistic insights into these clinical findings. Peritoneal mesothelial cells, which naturally produce nitric oxide (NO), exhibit enhanced NO synthesis upon exposure to L-arginine or AA-PD [60, 101]. In vitro, AA-PD approximately doubled nitrite levels relative to control media, an effect abolished by NO synthase inhibition—suggesting that the L-arginine in AA-PD solution acutely stimulates NO production, potentially contributing to the increased peritoneal perfusion observed in vivo [44, 59, 101].

Further evidence for the biocompatibility of Nutrineal PD4 is shown by markers of mesothelial cell viability. Changes in the morphology and phenotype of mesothelial cells are commonly observed during long-term PD. Alongside denudation of the mesothelial monolayer, these alterations are frequently seen in failing peritoneal membranes, supporting the notion that mesothelial-to-mesenchymal transition (MMT) plays a central role in membrane remodeling [102]. However, the contribution of MMT to peritoneal fibrosis remains somewhat controversial, as lineage-tracing experiments have not consistently supported its involvement [102].

Chan et al. conducted an ex vivo analysis comparing the impact of peritoneal dialysate collected after 4-hour exchanges using amino acid–based versus glucose-based PD solutions on human peritoneal MCs [55]. Their findings demonstrated that cellular ultrastructure and survival were more effectively maintained under amino acid exposure, and the suppression of cell proliferative capacity was less pronounced. However, the amino acid–based solution was associated with an upregulation of interleukin-6 (IL-6) secretion by the cultured human peritoneal MCs, suggesting a mild proinflammatory response [55].

Chang et al. [65] cultured human mesothelial cells derived from patients who substituted one daily exchange of glucose-based solution with AA-PD. After three months, effluent levels of cancer antigen 125 (CA125)—a biomarker of mesothelial cell mass - increased, suggesting improved cellular health and better biocompatibility than the conventional glucose-based PD solutions [65]. These findings are consistent with broader reviews documenting AA-PD’s favorable cellular profile [15–17, 32, 55, 65].

Additionally, an in vivo experimental study in rabbits demonstrated that replacing glucose with amino acids (AAs) as the osmotic agent in PD solutions prevented mesothelial injury and microvascular alterations [103]. The superior biocompatibility of AA-based dialysates is attributed to a lower glucose burden, thereby minimizing the generation of GDPs and AGEs, as well as to their more physiological pH, which reduces cellular stress and inflammation.

The use of glucose-free peritoneal dialysis fluids, particularly those based on AAs, appeared to support the preservation of MCs and maintain local immune competence [62]. Martikainen et al. reported that AA-based and, to a greater extent, icodextrin-based solutions may be associated with a modest activation of systemic and intraperitoneal inflammation, as indicated by elevated serum C-reactive protein and increased levels of IL-6 and tumor necrosis factor-alpha (TNF-α) in the dialysate [62]. This inflammatory response could reflect enhanced cellular integrity, allowing for a more active immune signaling environment. Supporting this, Brulez et al. observed superior preservation of peritoneal macrophage function with AA-based solution compared to conventional 2.27% glucose-containing solutions, highlighting the immunoprotective potential of glucose-sparing strategies [104].

While robust long-term structural data are still lacking, theoretical considerations suggest that reducing intraperitoneal glucose exposure may attenuate chronic peritoneal injury processes, including inflammation, fibrosis, and vasculopathy [105–110]. Amino acid–based solutions such as Nutrineal PD4 remove GDPs, recognized mediators of peritoneal damage, from the dialysate environment. In one clinical study assessing so-called “biocompatible” PD fluids (including AA–based options), peritoneal solute transport rates increased more gradually and plateaued by two years, in contrast to the continuous rise seen with conventional glucose-based solutions [111]. Notably, no clinical trial to date has linked the use of AA-PD solutions with accelerated deterioration of the peritoneal membrane [50, 97, 110].

In summary, current evidence indicates that amino acid–based peritoneal dialysis solutions preserve and may modestly enhance, peritoneal membrane function. Although its long-term impact on the histological change of peritoneum remains to be illustrated, the available clinical and mechanistic data support the relative biocompatibility of amino acid–based dialysates.

### Biochemical rationale behind amino acid–based peritoneal dialysis solutions

Protein-energy malnutrition/wasting (PEW) is a common comorbidity in CKD, affecting approximately 40% of patients undergoing dialysis [111]. Although reduced dietary intake of protein and energy is a primary contributor, PEW also arises from metabolic acidosis, insulin resistance, and the accumulation of uremic toxins [79, 112].

In PD, protein losses range from 5 to 10 g/day, with an additional 2 to 4 g/day of amino acid loss, even in non-infected patients [83–85]. Emerging evidence highlights systemic inflammation as a critical driver of malnutrition, with strong associations between inflammatory markers, poor nutritional status, and elevated cardiovascular mortality in dialysis populations [86–91].

#### Protein supplementation

Despite various strategies to improve nutritional intake in PD patients, actual protein consumption often remains below the recommended 1.2 g/kg/day. In CAPD, AA-enriched dialysates have been utilized to supplement insufficient dietary protein and offset peritoneal losses [30, 49, 54, 80, 113–115].

Each 2-L exchange of Nutrineal PD4 contains approximately 22 g of amino acids (equivalent to 3.8–4.5 g of nitrogen, given a nitrogen content of ~ 16%). A 2-L exchange of 1.1% AA solution typically delivers 15–18 g of amino acids over 6 h, with 40–80% being absorbed during a 4–6-hour dwell [47, 50, 116]. Thus, 1–2 exchanges daily can contribute substantially toward meeting the protein requirement of 1.2 g/kg/day. In malnourished PD patients, such supplementation has been shown to induce a positive nitrogen balance; for example, one controlled study demonstrated significant improvement in nitrogen retention with 1–2 Nutrineal PD4 exchanges per day [42, 50, 61, 116]. The number of AA exchanges should be tailored to the patient’s clinical status. While a single exchange is generally recommended, up to two may be considered in patients with low degree of azotemia and good tolerance. Severely malnourished patients may tolerate more; for instance, a study by Jones et al. (1998) found that approximately 60% of such patients tolerated two CAPD exchanges per day [49].

#### Anabolic effect

Intraperitoneal amino acid administration has been shown to promote whole-body anabolism [42]. Studies using stable isotope tracer techniques have confirmed that AA-PD, when combined with oral caloric intake, enhances protein synthesis and reduces proteolysis, mimicking the metabolic effects of dietary protein [30, 54, 108–112]. In a nocturnal APD protocol, the combined infusion of amino acids and glucose yielded a net protein gain of approximately 13 g per session, sufficient to offset typical protein losses [116].

Furthermore, current evidence indicates that amino acid–based peritoneal dialysis solutions are associated with reductions in serum phosphorus and with markers of an anabolic response, including increased insulin-like growth factor-1 levels and enhanced amino acid transport into skeletal muscle [72, 116, 117].

Clinical studies have reported improvements in serum albumin and other nutritional markers in malnourished or hypoalbuminemic PD patients treated with AA-PD [43, 49, 54, 61, 80, 113–115, 118–120]. A crossover study involving 18 CAPD patients receiving one daily postprandial 2-L Nutrineal PD4 exchange reported a significant rise in mean serum albumin among those with baseline levels < 30 g/L (from 26.8 to 30.1 g/L at 6 months, *p* < 0.01) [43]. Other benefits observed include increases in mid-arm muscle circumference and improved composite nutritional scores [120–122].

Although multiple factors influence serum albumin levels, both acute and chronic inflammatory states are particularly significant due to their impact on hepatic protein metabolism and increased capillary permeability, which collectively contribute to hypoalbuminemia [123].

In the context of PD, systemic inflammation may attenuate the effectiveness of protein-based nutritional interventions such as AA-PD in raising serum albumin concentrations [124–128]. Inflammatory processes not only suppress hepatic albumin synthesis but also enhance its degradation [125, 126]. Supporting this, a study in hemodialysis patients demonstrated a sustained decline in serum albumin levels associated with elevated inflammatory markers, specifically IL-6 and C-reactive protein (CRP), independent of changes in protein intake, suggesting that inflammation, rather than malnutrition, is a primary driver of hypoalbuminemia in this population [126].

Similarly, in malnourished patients undergoing CAPD, systemic inflammation was associated with a blunted response to nutritional supplementation. Patients with elevated levels of high-sensitivity CRP and IL-6 exhibited a less pronounced increase in serum albumin following oral egg albumin-based supplementation. While both inflamed and non-inflamed groups showed improvements in overall nutritional status, the non-inflamed group experienced a greater rise in serum albumin (from 3.0 ± 0.9 to 3.4 ± 1.1 g/dL, *p* = 0.08) compared to the inflamed group (from 2.8 ± 0.6 to 3.0 ± 0.9 g/dL, *p* = 0.66), underscoring the inhibitory effect of inflammation on albumin response [128].

### Clinical rationale behind amino acid–based peritoneal dialysis solutions

AA-PD solutions offer nutritional and metabolic advantages over conventional glucose-based solutions, particularly in maintaining or improving nutritional status in PD patients [61].

Short- and long-term studies have consistently demonstrated their anabolic effects.

In a controlled study, UF and small solute clearance over a 6-hour dwell with a 1% amino acid–bicarbonate solution were comparable to those achieved with 1.5% glucose–bicarbonate (Glu/Bic) and 1.5% glucose–lactate (Glu/Lac) solutions. Importantly, amino acid–bicarbonate solution reduced serum glucose levels and effectively compensated the protein-nitrogen loss equivalent to approximately three glucose dwells, while bicarbonate buffering (34 mmol/L) did not adversely affect systemic acid-base status [51].

Faller et al. [80] reported significant increases in serum albumin (from 32.7 to 35.1 g/L) and transferrin after three months of treatment with a 1.1% amino acid solution. Similarly, Jones et al. [49] observed increases in insulin-like growth factor 1 (IGF-1), prealbumin, transferrin, and mid-arm muscle circumference following one or two daily exchanges with AA-PD, changes not seen in patients receiving glucose-based dialysate.

Long-term benefits were confirmed by Taylor et al., who observed increased serum albumin (22.5 to 25.7 g/L; *p* = 0.0036) and normalized protein catabolic rate (nPCR) (from 0.90 to 1.09 g/kg/day) in hypoalbuminemic CAPD patients treated with AA-PD for a mean of 13.6 months, without significant changes in body weight or residual renal function [33]. Over a three-year period, Li et al. [54] found that daily AA-PD use helped preserve or improve serum albumin and cholesterol levels, whereas patients on glucose dialysate experienced declines. Nutritional markers such as protein intake and protein equivalent of nitrogen appearance also improved, particularly in women, who maintained lean body mass index (BMI) [50, 54]. In children, long-term use of amino acid/glucose solutions led to better growth outcomes [129, 130].

Beyond nutritional improvements, amino acid–based solutions reduce glucose exposure, offering metabolic benefits. By eliminating dialysate glucose absorption, these solutions support better lipid and glycemic profiles. Li PK et al. [131] reported reduced serum triglycerides with AA-PD, while the IMPENDIA/EDEN RCT (*n* = 251) showed that a low-glucose regimen (using both icodextrin and amino acid solutions) achieved a 0.5% greater reduction in HbA₁c and improvements in triglycerides, very low-density lipoprotein, and Apolipoprotein B compared to glucose-based PD [131]. However, a single AA-PD exchange may transiently raise plasma glucose in diabetic patients via gluconeogenesis [132].

Importantly, substituting one daily glucose exchange with the AA-PD solution does not compromise dialysis adequacy. In Li FK et al.’s RCT, weekly Kt/V (urea) and daily UF volumes remained equivalent between treatment groups [54]. Pediatric studies also reported comparable creatinine clearance and UF with amino acid– versus glucose-based solutions [133]. Furthermore, the AA-PD solution demonstrated a higher mass transfer area coefficient for creatinine and similar or better clearance of low- and high-molecular-weight solutes compared to 1.36% glucose dialysate [54, 120–122]. A single exchange with the AA-PD solution achieves an ultrafiltration profile similar to that of a 1.36% glucose solution [75, 134, 135]. Because of these properties it has been suggested that amino acid-based PD solutions should be considered as an integral part of glucose-sparing strategies [136].

In addition to metabolic benefits, amino acid–based solutions exhibit enhanced biocompatibility. Short-term exposure to conventional PD fluids impairs cytokine responses by activated leukocytes, contributing to peritoneal inflammation. Bicarbonate-buffered amino acid solutions preserve leukocyte function, indicating reduced proinflammatory activation compared with glucose-based fluids [99]. Likewise, human peritoneal mesothelial cells exposed to amino acid–bicarbonate solutions show lower heat shock protein (HSP)-72 induction, maintained viability, and preserved cytokine production. Basal release of IL-6 and prostaglandin E_2_ is also enhanced, reflecting preserved physiological function and a favorable biocompatibility profile [100].

Table 3 summarizes key studies comparing clinical and metabolic outcomes between amino acid– and glucose-based PD solutions. These results show that significant heterogeneity remains in literature about the effects of amino acid–based PD solutions on nutritional status even in patients who are malnourished or at high risk of becoming so. Furthermore, long-term data on technique survival and mortality with amino acid–based PD solutions are still lacking.

**Table 3:** Summary of key studies comparing amino acid–based versus glucose-based peritoneal dialysis solutions. The studies have been listed according to the year of publication

Author(s)	Comparator	Main outcomes evaluated	Key results
Douma et al.; 1996 [44]	1.36% glucose dialysate (Dianeal^®^)	Peritoneal transport, ultrafiltration, solute clearances, and MTAC	AA dwell increased peritoneal blood flow and MTACs for small solutes (creatinine, urea) and raised transcapillary ultrafiltration rate vs. glucose; net ultrafiltration similar due to higher lymphatic absorption.
Jones et al.; 1998 [49]	1.36% glucose dialysate (Dianeal^®^)	Nutritional indices (albumin, body weight, dietary intake), tolerability.	AA group (1–2 exchanges/day) showed improved amino-acid availability and some short-term nutritional benefits vs. glucose; tolerability acceptable but modest clinical impact on albumin/BMI.
Plum et al.; 1999 [51]	Glucose-bicarbonate and glucose-lactate solutions	Acute metabolic effects, peritoneal transport	AA solution produced different acute metabolic responses and transport characteristics vs. glucose solutions; demonstrated safety and distinct kinetics.
Taylor GS et al.; 2002 [33].	None	Serum albumin, normalized protein catabolic rate (nPCR), weight, Kt/V, CrCl, peritonitis rate, mortality	In 22 hypoalbuminemic CAPD patients (13.6 mo on Nutrineal), albumin increased significantly (mean 22.45 to 25.68 g/L, *P* = 0.0036) and nPCR increased (from 0.898 to 1.085 g/kg/d, *P* = 0.0057). Weight was stable. Kt/V and CrCl fell slightly but remained adequate in > 80%. Only 1 death (4%/yr) and low peritonitis were observed.
Li FK et al.; 2003 [54].	1.36% glucose dialysate (Dianeal^®^)	Nutritional markers (albumin, cholesterol, nPCR, dietary protein intake, body mass), dialysis adequacy, survival	In 60 malnourished CAPD patients followed 3 years, daily AA dialysate (*n* = 30) vs. glucose (*n* = 30) stabilized/increased nutritional parameters: serum albumin and cholesterol declined in controls but remained stable or rose with AA; Normalized protein intake and dietary protein intake increased only with AA; lean body mass (women) was preserved on AA. Total Kt/V and UF were maintained in both groups.
Tjiong HL et al.; 2005 [61]	Glucose-only dialysate	Protein metabolism (leucine kinetics, 24-h nitrogen balance), nutritional status	In a crossover study of 8 automated PD patients, one night exchange of 1.1% AA+glucose dialysate significantly increased net protein balance vs. glucose alone (mean increase 0.21 µmol leucine/kg/min, *P* < 0.001). Nitrogen balance trended positive with AA (*P* = 0.06). Conclusion: combining AA with glucose dialysate enhances protein anabolism compared to glucose alone.
Dervisoglu E et al.; 2010 [66]	1.36% glucose dialysate (Dianeal^®^)	Serum albumin, total protein	Retrospective study of 32 incident CAPD patients over 12 months found no significant benefit of AA dialysate on nutrition compared with 32 CAPD patients using dextrose solution. Baseline albumin was lower in the AA group, but at 1 year neither group showed significant changes in albumin or total protein.
Li PKT et al. [IMPENDIA/EDEN Trial]; 2013 [132]	1.36% glucose dialysate (Dianeal^®^)	Glycemic control (HbA1c), lipid profile (TG, VLDL, ApoB), fluid volume and adverse events	In 251 diabetic PD patients (180 in IMPENDIA and 71 in EDEN) randomly assigned to intervention (*n* = 124) or control (*n* = 127) group, a glucose-sparing regimen (dextrose + icodextrin + amino acids) improved metabolic control: HbA1c fell ~ 0.5% more than control, and triglycerides/VLDL/ApoB decreased. The AA/icodextrin arm had more volume-overload events and deaths.
Iyasere et al. [Metanalysis]; 2022 [72]	AAD versus standard solutions (various)	Anthropometrics and serum albumin (pooled evidence)	AA solutions can improve protein kinetics / some anabolic markers, but pooled effects on albumin and anthropometrics were inconsistent and of limited quality. The benefit may be limited to selected malnourished patients; evidence not definitive.

MTAC: Mass transfer area coefficient; AA: Amino acid; BMI: Body mass index; ApoB: Apolipoprotein B; CAPD: Continuous ambulatory peritoneal dialysis; CrCl: Creatinine clearance; HbA1c: Glycated hemoglobin; Kt/V: Dialysis adequacy index for urea clearance; nPCR: Normalized protein catabolic rate; PD: Peritoneal dialysis; TG: Triglycerides; UF: Ultrafiltration; VLDL: Very low-density lipoprotein; AAD: Amino-acid dialysate

### Clinical safety and metabolic considerations of amino acid-based dialysates in peritoneal dialysis

The safety profile of amino acid-based PD solutions has been evaluated across multiple clinical studies. Nutrineal PD4 has demonstrated good tolerability in both adult and pediatric trials. However, the primary concerns associated with its use include the potential for metabolic acidosis and nitrogenous waste accumulation, particularly when used more than once per day. Consequently, due to the risks of acidosis and azotemia, current recommendations advise limiting its use to a single peritoneal exchange per day [137].

Additionally, amino acid–based PD solutions should ideally be administered in conjunction with a substantial meal or simultaneous glucose instillation during APD, to promote efficient protein synthesis and minimize the conversion of amino acids into urea, thereby preventing excessive elevations in blood urea nitrogen (BUN) [76, 77].

Newer biocompatible PD fluids, such as Physioneal^®^, have been formulated at neutral pH with low GDP content. These properties have been associated with improved in vitro biocompatibility and better preservation of residual renal function in both experimental and clinical studies [138, 139]. Nutrineal PD4 differs fundamentally from conventional glucose-based solutions because it is an amino acid–based, glucose-free dialysate and therefore does not generate GDPs that are centrally implicated in AGE formation and GDP-mediated peritoneal injury [30–32]. Although its nominal pH is lower than that of Physioneal, Nutrineal PD4 is typically prescribed intermittently (e.g., one exchange per day), thereby limiting cumulative exposure. Several clinical investigations have demonstrated that the AA-PD solution provides net systemic amino acid uptake and can improve protein kinetics and nutritional indices in selected patients [33, 44, 49, 54, 61, 66, 72, 132]. Thus, the slightly lower pH of Nutrineal PD4 should be considered alongside its absence of glucose/GDP exposure; in practice, Nutrineal and neutral-pH low-GDP fluids may be viewed as complementary therapies with distinct indications, potentially offering synergistic benefits when used in combination.

In a 3-month clinical trial involving pediatric patients, amino acid-based dialysis did not result in any clinical adverse events or significant worsening of metabolic acidosis [133]. As anticipated, the principal metabolic effect observed was an elevation in BUN, attributed to amino acid catabolism [133]. Mild hyperammonemia is an uncommon finding with modern formulations. Rarely, patients may experience transient side effects such as nausea or hypotension.

Faller et al. reported that, although serum urea levels increased as expected, serum bicarbonate levels remained stable after three months of treatment with amino acid-based solutions [80]. Similarly, Jones et al. conducted a 12-week study demonstrating that these solutions are both safe and well tolerated [49]. In a longer-term study by Li FK et al., spanning three years, the use of amino acid-containing dialysate did not compromise dialysis adequacy or the peritoneum’s ultrafiltration capacity [54].

Metabolic concerns associated with AA-PD (e.g., azotemia and mild acidosis) contrast with those linked to glucose-based dialysates, which carry risks such as hyperglycemia, weight gain, and peritoneal fibrosis due to exposure to glucose degradation products [32, 44, 110, 134].

The incidence of peritonitis in patients using AA-PD appears equivalent to the incidence observed with conventional PD fluids. In a retrospective cohort of 22 CAPD patients receiving AA-PD, Taylor et al. observed that AA-PD solutions could be used safely and effectively for extended periods (duration of use 6–26 months), and such use was associated with a low peritonitis rate and a low mortality rate [33]. A larger comparative study by Duranay et al. (2007) involving 147 CAPD patients found no statistically significant difference in peritonitis rates between those using glucose-only dialysate, glucose combined with icodextrin, and glucose combined with AA-PD [63]. The sole independent predictor of peritonitis in this study was hypoalbuminemia (serum albumin < 3 g/dL), rather than the type of dialysate used [63].

The use of the AA-PD appears not to be associated with unusual peritonitis pathogens. Most infections observed in clinical use are attributable to common skin flora, such as coagulase-negative staphylococci, like those seen in standard CAPD. Thus, no data currently suggests that AA-PD increases infection risk or alters microbiological patterns [33, 63].

In conclusion, amino acid-based PD solutions are generally safe and well tolerated. While no severe toxicities have been reported, standard clinical monitoring (e.g., fluid status, serum electrolytes) is essential. Nutrineal PD4 is contraindicated in patients with severe metabolic acidosis or hepatic failure due to its limited buffering capacity.

### Peritonitis and stability of antimicrobial agents in PD solutions: implications for intraperitoneal therapy

PD-associated peritonitis remains a major clinical concern, contributing significantly to patient morbidity and mortality, with outcomes largely influenced by the causative pathogen [140, 141]. Recurrent or prolonged episodes of peritonitis may induce irreversible alterations to the peritoneal membrane, ultimately necessitating transition to hemodialysis [142, 143]. As emphasized by the International Society for Peritoneal Dialysis (ISPD) guidelines, early initiation of empiric intraperitoneal (IP) antimicrobial therapy with solutions permitting a minimum dwell time of 6 h is essential for optimal management of this complication [144].

Current evidence suggests that the composition of dialysis solutions may influence peritonitis risk by modulating host immune responses and exerting direct effects on peritoneal integrity [26, 141].

A comprehensive review by So et al. addressed the stability profiles of frequently used antimicrobials, such as penicillins, cephalosporins (e.g., cefazolin, ceftazidime, cefepime), vancomycin, gentamicin, ciprofloxacin, and carbapenems (imipenem, meropenem), in PD fluids [145]. However, data remain limited regarding the compatibility of less commonly utilized antibiotics, particularly in newer neutral-pH, low-GDP solutions presented in multi-compartment containers [73, 146]. Although pharmacokinetic data for many of these agents are currently lacking, comprehensive stability assessments can facilitate their use in clinical settings as pharmacokinetic insights emerge.

The stability of various antimicrobial agents has been investigated in the amino acid–based peritoneal dialysis solution formulated with a more biocompatible pH compared to conventional acidic glucose-containing non-biocompatible solutions [57].

Aminoglycosides such as gentamicin and netilmicin exhibit robust stability in the amino acid–based peritoneal dialysis solution, maintaining chemical integrity for a minimum of 24 h at ambient temperature (25 °C) and an additional 4 h at physiological temperature (37 °C) [55]. Vancomycin, a glycopeptide antibiotic, demonstrates similar stability parameters under the same conditions [57]. Among β-lactams, cefazolin and ceftazidime preserve over 90% of their initial concentrations for clinically relevant periods (24 h at 25 °C, 4 h at 37 °C) when dissolved in Nutrineal PD4 [57]. Furthermore, stability data for less routinely employed agents such as aztreonam and daptomycin are encouraging: aztreonam retains over 90% of its initial potency for up to 7 days under refrigeration (4 °C) [71], and daptomycin demonstrates sustained stability for 7 days at 4 °C, with additional in-use stability for at least 6 h at 37 °C [147].

These findings substantiate the suitability of amino acid–based peritoneal dialysis solution as a vehicle for a broad spectrum of IP antimicrobials, with stability profiles that support both continuous ambulatory and automated peritoneal dialysis regimens.

Table S1 (Supplemental material) provides a summary of the stability profiles of various antibiotics across different peritoneal dialysis solutions.

### Clinical considerations for the use of amino acid-based solutions and future perspectives

To facilitate the translation of these findings into clinical practice, the following opinions are proposed for the targeted use of amino acid-based PD solutions:

#### Patient selection and prioritization

Use is recommended especially for patients with evidence of protein-energy wasting or those at high risk of malnutrition who are unable to achieve an adequate protein intake (1.0–1.2 g/kg/day) through diet or oral supplements.

#### Dosage and administration

In clinical practice, in a patient with body weight 70 kg and no apparent contraindications such as clinical or laboratory signs of increasing severity of uremia such as serum urea > 38 mmol/liter or metabolic acidosis such as serum bicarbonate < 18 mmol/l, use is restricted to one daily exchange of 2–2.5 L of a 1.1% amino acid solution. This is typically sufficient to provide approximately 25% of the daily protein requirement. To maximize protein synthesis, this exchange should ideally coincide with a high-carbohydrate meal.

#### Monitoring

Clinicians should monitor plasma urea and bicarbonate levels. Similar to the metabolic effects of an increased dietary protein intake, an increase in plasma urea levels and a decrease in bicarbonate levels are expected and generally indicates amino acid utilization; however, excessive increases may require dose adjustment or temporary discontinuation.

#### Logistical constraints

While amino acid solutions are a valuable tool in the nutritional armamentarium, factors such as local availability and cost-effectiveness should be considered. The implementation of amino acid-based solutions in routine clinical practice is often influenced by economic factors. These specialized solutions carry a higher acquisition cost compared to standard glucose-based dialysis fluids. The extra cost of using the AA-PD solution - and a “triple bag” regimen combining AA-PD, icodextrin and glucose-based normal pH and low-GDP solutions could not be afforded by patients in most low and low-medium income countries. This poses an obvious challenge to the equity of access to care.

However, a comprehensive assessment of value should consider the potential for these solutions to mitigate the high costs associated with protein-energy wasting (PEW), such as increased hospitalization rates and infection-related complications. Targeted prescriptions for the most vulnerable patient cohorts may optimize the cost-benefit ratio, though formal health-economic evaluations in various healthcare systems are still warranted [11, 112, 127]. Emerging data also suggests a potential role in preserving residual kidney function, particularly when used as part of a comprehensive glucose-sparing strategy.

Two ongoing clinical trials (NCT07077538 [148] and NCT06597201 [149]) in China are evaluating an AA-PD solution (Chengdu Qingshan Likang Pharmaceutical Co., Ltd), focusing on glycemic control in diabetic patients and serum albumin improvement in PD patients with malnutrition, respectively. The objectives of these two studies are to observe changes of glycosylated hemoglobin relative to baseline 90 days after using the amino acid PD solution in diabetic patients and to provide evidence on the efficacy and safety of the amino acid PD solution in PD patients with malnutrition [150]. Another study in China evaluates the efficacy and safety of AA-PD solution in improving nutritional status and clinical outcomes among malnourished PD patients [150].

Beyond standard regimens, amino acid-based solutions are increasingly being integrated into more complex prescriptions, such as the ‘three-bag’ protocol. This approach—combining one exchange each of amino acids, glucose, and icodextrin—is gaining attention as a potential method for prescribing incremental PD. By leveraging the different osmotic and nutritional properties of these three solutions, clinicians may be able to further minimize glucose exposure while supporting nutritional status. Another approach to reducing the effects of intraperitoneal glucose exposure is to implement an incremental PD strategy with a reduced frequency of exchanges with glucose-based solutions for the short dwells and if needed using the icodextrin-based solution for the long dwell.

While an in-depth objective analysis of the clinical efficacy and safety of the three-bag or two-bag protocol is the subject of a separate publication, its role in personalized, glucose-sparing dialysis warrants mention as an evolving area of clinical practice [11].

This review expanded upon previous systematic reviews and meta-analyses by incorporating recent longitudinal evidence and mechanistic data. Whereas earlier quantitative syntheses primarily focused on nutritional parameters, such as serum albumin, our review additionally evaluated the long-term effects on peritoneal membrane integrity and residual kidney function. Moreover, the application of the SWiM framework enabled the structured synthesis of heterogeneous evidence into clinically relevant considerations. This approach provided a more comprehensive perspective on the role of amino acid-based solutions within contemporary glucose-sparing and incremental PD strategies.

### Study limitations

A primary limitation of this systematic review is that the data were synthesized qualitatively, without a formal assignment of levels of evidence or a structured cross-study assessment of certainty (e.g., via the Grading of Recommendations Assessment, Development, and Evaluation [GRADE] framework). While this descriptive approach was necessitated by the marked heterogeneity of the included clinical trials and mechanistic studies, it inherently limits the capacity to definitively stratify the comparative strength of the resulting clinical recommendations.

Although the compiled evidence suggests that amino acid-based solutions offer potential utility for nutritional optimization, several critical caveats must be addressed. First, the majority of available trials relied primarily on surrogate biochemical markers—such as serum albumin levels or nitrogen balance—rather than hard clinical endpoints, including technique survival or overall mortality. Second, substantial methodological variability across study protocols precludes the formulation of a universal standard of care. Third, most of the studies included and commented on are old and thus compare the AA-PD solution to a “conventional” acidic glucose-based PD solution with high GDPs and not to glucose-based PD solutions with normal pH and low concentration of GDPs. Finally, given the potential for bias in industry-funded literature or studies involving investigators with corporate affiliations, there is a clear imperative for independent, large-scale, multicenter randomized controlled trials.

Such robust research is essential to confirm whether these surrogate nutritional improvements translate into meaningful, long-term clinical advantages for the peritoneal dialysis population. Meanwhile, rather than aiming for studies on hard endpoints such as time on therapy or patient survival which would require large, multicenter, independent RCTs long enough to let the events occur, other endpoints should be considered such as the efficacy of AA-PD solutions in mitigating the ominous gradual substitution of lean body mass with fat tissue mass in long-term PD.

## Conclusion

This systematic review highlights the therapeutic potential of amino acid-based PD solutions in addressing protein-energy wasting by providing an alternative nitrogen source and while at the same time reducing glucose exposure. The evidence synthesized suggests that one daily exchange of a 1.1% amino acid solution is generally well-tolerated and effective in improving nutritional surrogate markers. From a clinical management perspective, a significant limitation of amino acid-based solution administration involves the risk of metabolic acidosis and the accumulation of nitrogenous waste products, particularly when utilizing multiple daily exchanges. Consequently, to mitigate the potential for severe acid-base disturbances and progressive azotemia, current clinical practice guidelines recommend restricting its use to a single peritoneal dwell per day.

However, significant heterogeneity remains in the literature, and long-term data on technique survival and mortality are still lacking. Targeted use of these solutions, particularly in malnourished or high-risk patients, represents a personalized approach to optimizing peritoneal dialysis outcomes. Future multicenter trials are necessary to refine clinical protocols and establish long-term efficacy.

## Supplementary Information

Below is the link to the electronic supplementary material.

Supplementary Material 1

## Data Availability

The data supporting the findings of this systematic review were sourced from previously published peer-reviewed articles, which are cited throughout the manuscript and listed in the reference section. All data included in this systematic review were extracted from previously published studies identified through the systematic literature search. No individual participant data were collected or generated for this study. All extracted data derives from publicly available sources (peer-reviewed articles and conference publications) and are reported within the manuscript and supplementary materials, where applicable.

## Abbreviations

AA-PD: Amino acid–based peritoneal dialysis
AGEs: Advanced glycation end products
APD: Automated peritoneal dialysis
BMI: Body mass index
BUN: Blood urea nitrogen
CA125: Cancer antigen 125
CAPD: Continuous ambulatory peritoneal dialysis
CCPD: Continuous Cycling Peritoneal Dialysis
CKD: Chronic kidney disease
CO₂-MTAC: Carbon dioxide mass transfer area coefficient
CRP: C-reactive protein
D/P₍Cr₎: dialysate-to-plasma creatinine ratio
EMT: Epithelial -to-Mesenchymal Transition
GDP: Glucose degradation products
3-DG: 3-deoxyglucosone
Glu/Bic: Glucose–bicarbonate
Glu/Lac: Glucose–lactate
HD: Hemodialysis
HSP: Heat shock protein
IGF-1: Insulin-like growth factor 1
IgG: Immunoglobulin G
IL: Interleukin
IP: Intraperitoneal
ISPD: International Society for Peritoneal Dialysis
MC: Mesothelial cell
MMT: Mesothelial-to-mesenchymal transition
NO: Nitric oxide
nPCR: Normalized protein catabolic rate
PD: Peritoneal dialysis
PET: Peritoneal equilibration test
PEW: Protein-energy malnutrition/wasting
PM: Peritoneal membrane
PRISMA: Preferred Reporting Items for Systematic Reviews and Meta-Analyses
PSTR: Peritoneal solute transfer rate
RAGE: Receptor of advanced glycation end products
RCTs: Randomized controlled trials
RKF: Residual kidney function
RWE: Real-world evidence
SWiM: Synthesis Without Meta-analysis
TNF-α: Tumor necrosis factor-alpha
UF: Ultrafiltration
