# Supplementary material for: Therapeutic potential of amino acid-based peritoneal dialysis solutions: a systematic review
Source: BMC Nephrol. 2026 Jul 30;27:449. doi: 10.1186/s12882-026-05222-3 (PMC13428450; doi:10.1186/s12882-026-05222-3)
Supplement: Supplementary file 1 — Supplementary Material 1 [file 12882_2026_5222_MOESM1_ESM.docx]

**Supplementary Material**

Table S1. Overview of Antibiotic Stability in Various Peritoneal Dialysis (PD) Fluids. Adapted from Ling et al. [73].

| **Antibiotic Agent** | **Glucose-Based Dialysates** | **Icodextrin (Extraneal)** | **Amino Acid-Based (Nutrineal)** | **Neutral pH, Low-GDP Solutions** |
| --- | --- | --- | --- | --- |
| **Agents with validated stability suitable for clinical use** |  |  |  |  |
| *Intermittent Dosing* |  |  |  |  |
| Cefotaxime | ✓^a^ | – | – | – |
| Ceftriaxone | ✓ ^b^ | – | – | – |
| Fosfomycin | – | - | ✓^1^ | ✓^c^ |
| Tobramycin | ? | ✓ | – | ? |
| *Continuous Dosing* |  |  |  |  |
| Daptomycin | – | – | ✓^2^ | ✓^d^ |
| Ofloxacin | ✓^b^ | – | – | – |
| Teicoplanin | ✓^e^ | – | – | – |
| **Agents with limitations in tested dose/concentration** |  |  |  |  |
| Aztreonam | Tested concentration supports only loading dose—not maintenance or intermittent use |  |  |  |
| Clindamycin | Concentration assessed was 1.5–2 times lower than ISPD-recommended dosage |  |  |  |
| **Agents not currently endorsed for intraperitoneal (IP) use by ISPD guidelines** |  |  |  |  |
| Amphotericin B (conventional) | ? | – | – | – |
| Amphotericin B (liposomal) | ✓^f^ | – | – | – |
| Anidulafungin | ✗ | ✗ | – | – |
| Ceftolozane/tazobactam | ✓^g^ | – | – | ✓^h^ |
| Cotrimoxazole | ? | – | – | – |
| Erythromycin lactobionate | ✓^i^ | – | – | – |
| Linezolid | – | ✓ | ✓^1^ | ✓^j^ |
| Moxifloxacin | ✓^k^ | – | – | – |
| Tigecycline | ✓^l^ | ✓ | – | ✓^m^ |

**Legend:**

✓ = Stable under tested conditions
✗ = Degradation observed (unstable)
– = Data not available/study not performed
? = Stability findings not generalizable to clinical use

**Footnotes (Solution Details):**

^1^Stable in Nutrineal PD4 for up to 14 days when refrigerate (See reference 146).

^2^Stable in Nutrineal for up to 7 days when refrigerate (See reference 146).

^a^Dianeal PD-1 (1.5% & 4.25% glucose); CAPD/DPCA ANDY disc 2 (1.5%) and disc 4 (2.3% glucose)

^b^Dianeal PD (1.5% & 4.25% glucose)

^c^Physioneal (1.36% & 2.27% glucose), tested both mixed and unmixed

^d^Balance solution (1.5% glucose), mixed and unmixed forms

^e^Dianeal PD-2 (1.5% glucose); applicable to continuous maintenance dosing (20 mg/L)

^f^ABLC (amphotericin B lipid complex) tested at 0.5 mg/L (Dianeal PD-1 1.5%), 2 mg/L (4.25%), and 10 mg/L (both 1.5% & 4.25%)

^g^Dianeal (1.5% & 2.5% glucose)

^h^Balance (1.3% & 2.3%) and Physioneal (1.36%, 2.27%, 3.86%), both mixed and unmixed

^i^Dianeal PD-1 (4.25% glucose)

^j^Physioneal 40 (1.36% & 2.27% glucose), tested both pre- and post-mixing

^k^Dianeal PD-1 (1.36% & 3.86% glucose)

^l^Dianeal (1.5% glucose)

^m^Balance (1.5% glucose), both mixed and unmixed
